# Elevated developmental temperatures impact the size and allometry of morphological traits of the bumblebee *Bombus terrestris*

**DOI:** 10.1242/jeb.245728

**Published:** 2023-04-19

**Authors:** Maxence Gérard, Marie Guiraud, Bérénice Cariou, Maxime Henrion, Emily Baird

**Affiliations:** ^1^ INSECT Lab, Division of Functional Morphology, Department of Zoology, Stockholm University, Svante Arrhenius väg 18b, 11418 Stockholm, Sweden; ^2^ Sorbonne Université, Faculté des Sciences et Ingénierie, 5 place Jussieu, 75005 Paris, France; ^3^ Ecole Normale Supérieure de Lyon, 15 parvis René Descartes, 69342 Lyon, France

**Keywords:** Antenna, Body size, *Bombus terrestris*, Global warming, Sensory traits, Wing

## Abstract

The impact of global warming on wild bee decline threatens the pollination services they provide. Exposure to temperatures above optimal during development is known to reduce adult body size but how it affects the development and scaling of body parts remains unclear. In bees, a reduction in body size and/or a reduction in body parts, such as the antennae, tongue and wings, and how they scale with body size (i.e. their allometry) could severely affect their fitness. To date, it remains unclear how temperature affects body size and the scaling of morphological traits in bees. To address this knowledge gap, we exposed both males and workers of *Bombus terrestris* to elevated temperature during development and assessed the effects on (i) the size of morphological traits and (ii) the allometry between these traits. Colonies were exposed to optimal (25°C) or stressful (33°C) temperatures. We then measured the body size, wing size, antenna and tongue length, as well as the allometry between these traits. We found that workers were smaller and the antennae of both castes were reduced at the higher temperature. However, tongue length and wing size were not affected by developmental temperature. The allometric scaling of the tongue was also affected by developmental temperature. Smaller body size and antennae could impair both individual and colony fitness, by affecting foraging efficiency and, consequently, colony development. Our results encourage further exploration of how the temperature-induced changes in morphology affect functional traits and pollination efficiency.

## Teaser

**Summary:** Bumblebees show a reduction in body size and antenna length and a change in allometric scaling of the tongue with elevated developmental temperature, indicating a strong impact of temperature on morphological traits.

## INTRODUCTION

Among ecosystem services, pollination is one of the most crucial for agricultural production and food security (IPBES, 2016) but, over the past few decades, wild pollinator populations have declined (Koh et al., 2016; Powney et al., 2019). Climate change is having an increasing impact on these pollinators, especially on wild bees (Gérard et al., 2020; Soroye et al., 2020). Increasing ambient temperatures can be problematic for bees as body temperature impacts many characteristics, from the rate of the biochemical processes of cells to ecological traits and behaviour, such as foraging and voltinism (Kingsolver and Huey, 2008; Gérard et al., 2022a). The relationship between temperature and development is also crucial for insects because it can impact the resulting phenotype, which ultimately affects function. Body size tends to decrease with increasing developmental temperature although, among bees, this relationship has only been observed in laboratory conditions (i.e. the temperature–size rule, TSR; Kingsolver and Huey, 2008; Radmacher and Strohm, 2011) and is less clear in the wild (Gérard et al., 2018a; 2021; Chole et al., 2019; but see Osorio-Canadas et al., 2016). Reduced body size can influence foraging behaviour, mostly by decreasing foraging range (Greenleaf et al., 2007; Kendall et al., 2019) but also pollen load capacity (e.g. Ramalho et al., 1998).

While previous literature has mostly focused on the effects of increasing temperature on overall body size, it could also affect the size of other traits that are important for pollination behaviour. For example, antennae are important organs for detecting floral resources and temperature variation (Yokohari, 1999; Ai et al., 2007). Antennal length can, in particular, affect olfactory sensitivity – shorter antennae are likely to have fewer receptors and this would reduce sensitivity (Spaethe et al., 2007). Additionally, wing size and shape are crucial for flight. Modification of wing morphology could affect flight parameters such as speed and acceleration, as observed in other insect species (Arambourou et al., 2017; Fraimout et al., 2018), and this would impact pollination efficiency. Finally, tongue length is related to the type of floral resources that can be accessed, and changes in tongue morphology can affect flower handling time and foraging efficiency (Klumpers et al., 2019). Despite the clear evidence that optimizing the morphology of these traits is of primary importance, how variations from optimal developmental temperatures affect adult morphology has only been addressed in a few studies. Among these studies, Gérard et al. (2018b) highlighted that wing size of bumblebee males could be reduced when exposed to elevated developmental temperatures in laboratory conditions, while Miller-Struttmann et al. (2015) showed that, with warming climate, the tongue length of bumblebees could decrease, leading to a functional mismatch with flower corolla depth that could threaten specialist species (Burkle et al., 2013; Miller-Struttmann et al., 2015).

Although previous work in bees has focused on how elevated developmental temperatures affect specific morphological traits, such as wings and tongue length, it remains unclear how different body parts are affected. As organs vary in their sensitivity to temperature during genesis (Vea and Shingleton, 2020), exposure to elevated temperatures during development is likely to have varying effects on different body parts. Allometry – defined here as how the size of a morphological trait scales with body size – is a way to explore whether and how organ genesis differs with variations in developmental temperature. By measuring the allometry of different morphological traits, we can gain a deeper understanding of how the developing organism has favoured or invested in different body parts, as well as how tightly constrained their sizes are. An animal cannot invest the same amount of energy into every trait and some traits may not be functional unless they are a particular size. The resulting trade-offs generate diversity in the ratio between their size and body size (Agrawal, 2020). Temperature deviations during development can affect this investment (Vea and Shingleton, 2020). For example, in *Drosophila melanogaster*, the cell proliferation of the wing imaginal discs is less sensitive to developmental temperature than the cell proliferation of the leg imaginal disc, leading to different growth rates of different morphological traits under different developmental temperatures (McDonald et al., 2018).

In this study, we assessed the impact of developmental temperatures on the size of the different morphological traits (i.e. body size, wing size, tongue and antennal length) of the buff-tailed bumblebee, *Bombus terrestris*, and on the allometry between these traits. Changes in these morphological traits and in their relative investment will ultimately determine how bumblebees perceive their environment, but also their efficiency to perform different tasks linked to the fitness of their colony. Indeed, these four morphological traits are particularly crucial for efficient foraging (i.e. body size and tongue length; Kendall et al., 2019; Klumpers et al., 2019), for detecting floral resources (i.e. antenna length; Spaethe et al., 2007) and flying to them (i.e. wing morphology; Mountcastle et al., 2016), but also for mating success (i.e. body size; Paxton, 2005). We measured these morphological traits in males and workers that underwent the entirety of their development in colonies kept either at 25°C, which is around the optimal temperature for colony growth and a temperature commonly experienced by bumblebees in temperate climates (Vogt, 1986; Weidenmüller et al., 2002; Nasir et al., 2019) or at 33°C, which is around the limit at which bumblebees start to increase fanning substantially and can be stressful for the colony (Vogt, 1986; Weidenmüller et al., 2002; Grad and Gradisek, 2018). We hypothesized that bees reared at 33°C would develop smaller body parts and that the size of these body parts relative to body size would also be smaller. We hypothesized that there would be differences in the allometry of the measured morphological traits between the castes, as they experience different selective pressures (i.e. foraging and brood care for workers, producing a new generation for males) and may exhibit differences in their capacity to buffer stressors during development.

## MATERIALS AND METHODS

### Experimental design

We obtained *Bombus terrestris* (Linnaeus 1758) colonies from Koppert B.V. company (Berkel en Rodenrijs, The Netherlands) and reared them in the dark at 50% humidity, in incubators (Panasonic MIR, 123 l) at the Department of Zoology, Stockholm University. Bumblebees were fed *ad libitum* with a 40:60 sugar–water solution, and fresh-frozen organic pollen every 2–3 days (Naturprodukter, Raspowder Bipollen). The experiments were conducted during two experimental sessions: from October to December 2020, and from January to March 2021. In total, eight colonies were reared at 25°C and eight colonies were reared at 33°C. After 25 days of development, all individuals in each colony were marked. Thus, at day 26, every newly emerged individual had experienced the full temperature treatment during its development, as 25 days corresponds to the duration of worker development (Duchateau and Velthuis, 1988). In total, we gathered a dataset of 347 workers (*n*=183 at 25°C, *n*=164 at 33°C) and 120 males (*n*=47 at 25°C, *n*=73 at 33°C).

### Morphological traits

We measured body size using intertegular distance (ITD, i.e. the distance between the two insertion points of the wing), a proxy often used for bumblebees (Cane, 1987), using a Cocraft 150 mm digital calliper (Insjön, Sweden). We used micro-scissors to clip off the tongue, as well as the right forewings and antenna of each bumblebee. Body parts that were damaged during this process were excluded from the analysis (see Table S1 for a summary of the dataset per trait). Morphological traits were photographed using a Leica Wild M3Z microscope (Wetzlar, Germany) coupled with a Canon EOS 70D camera (Tokyo, Japan). We used ImageJ (Schneider et al., 2012) to measure the antennae (the length of the flagellum and pedicel) and tongues (the length of the glossa). To calculate the size of the wing, we digitized each right forewing using two-dimensional cartesian coordinates of 18 landmarks using tps-DIG v2.32 (Rohlf, 2006; Fig. S1), which captures the shape and absolute size of a morphological trait through the manual placement of landmarks and calibration using objects of known size in the picture. The landmark configurations were then superimposed using the Generalized Procrustes Analysis superimposition (Bookstein, 1991; geomorph package: Adams and Otarola-Castillo, 2013). We calculated the centroid size of each wing – the square root of the sum of the squared distance between each landmark and the centroid of landmark configuration (Bookstein, 1991) – which is a proxy for wing size (Gérard et al., 2018b).

### Statistics

First, we assessed whether significant differences in ITD, antennal length, tongue length and wing centroid size were observed between males and workers reared at 25 and 33°C. To do so, for each trait, we built linear mixed models (LMM) after checking assumptions, using the lmer4 R package. These models were distinct for males and workers as their morphological traits differed significantly. When these assumptions were not verified even after trying several transformations of the dependent variable, we built generalized linear mixed models with a Gamma distribution (GLMM) using the lmer4 R package. Gamma distributions are adapted for non-normal positive and continuous data. We fitted different models with the size of the traits as the response variable and included temperature as a fixed effect, as well as colony and session as random effects. The final model was selected using the lowest AICc across all possible model combinations (which always included temperature treatment). We tested the different models against each other and selected the model with the lowest ΔAICc. If the ΔAICc was <2, the simplest model was selected. This last step was also included in the analyses described in the following paragraph. In addition, for each morphological trait and within each caste, we assessed whether any differences in the trait variance could be attributed to temperature, using *F*-tests (var.test function; package stats).

We then explored the allometric scaling relationships between ITD (as a proxy of body size) and the three other morphological traits (i.e. antennal length, tongue size and wing centroid size). We assessed whether the relationship between the morphological traits and ITD was isometric (i.e. the proportion between the morphological trait and ITD remains the same when ITD increases), hypoallometric (i.e. the morphological trait becomes proportionally smaller when ITD increases) or hyperallometric (i.e. the morphological trait becomes proportionally larger when ITD increases). We also assessed whether these allometric relationships changed depending on caste and developmental temperature, using the same statistical procedure (i.e. LMM or GLMM) described above. We built separate linear models for each sex and morphological trait. We fitted the models with the log_10_(size) of the traits as the response variable, log_10_(ITD) and the interaction between log_10_(ITD) and temperature as fixed effects, and colony and session as random effects. All the statistical analyses were computed using R Statistics.

## RESULTS

### The effect of developmental temperature on morphological traits

**Fig. 1.**
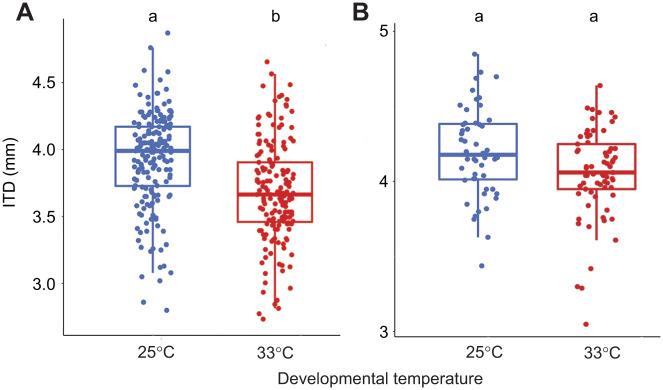
**Impact of developmental temperature on bumblebee body size.** (A) Intertegular distance (ITD; a proxy for body size) of workers (*n*=347; linear mixed model, LMM; *P*=0.006) and (B) ITD of males (*n*=120; LMM; *P*=0.53) raised at 25 or 33°C. Different letters at the top of the boxplots indicate significant differences. Each dot represents the measurement for one individual. First, second and third horizontal bars represent, respectively, the first quartile, the median and the third quartile. The two vertical bars represent the minimum and maximum, without considering the outliers.

The model that best explained variation in ITD included temperature and colony for workers (ΔAICc=1.34 with the next best candidate model, Table S2; *r*^2^=0.28, Table 1) and for males it included temperature and colony (*r*^2^=0.27, Table 2; ΔAICc=2.18 with the next best candidate model, Table S3). Developmental temperature had a significant effect on worker ITD, with workers reared at 33°C being significantly smaller (*P*=0.02; Fig. 1A), but had no significant effect on male ITD (*P*=0.53; Fig. 1B). The random factor ‘colony’ explained 17.8% of the variance that remained in the residuals for the workers while it explained 25.7% of the variance that remained in the residuals for the males. Variance in the ITD of males and workers was not significantly affected by the temperature treatment (*P*=0.74 and *P*=0.4, respectively).

**Table 1.**
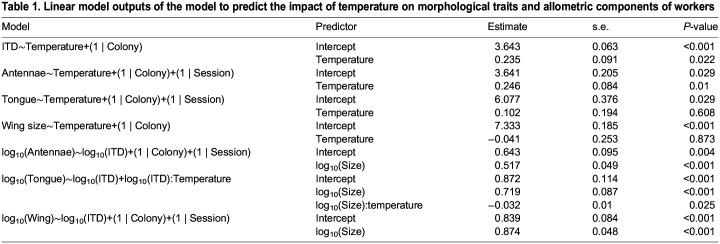
Linear model outputs of the model to predict the impact of temperature on morphological traits and allometric components of workers

**Table 2.**
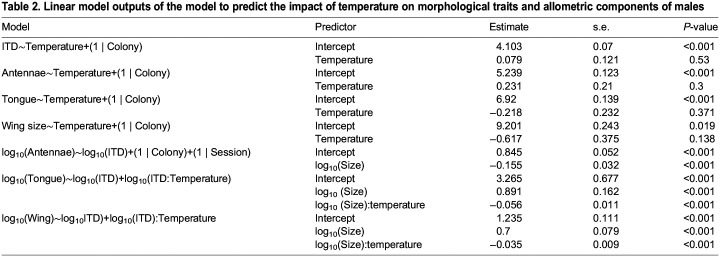
Linear model outputs of the model to predict the impact of temperature on morphological traits and allometric components of males

**Fig. 2.**
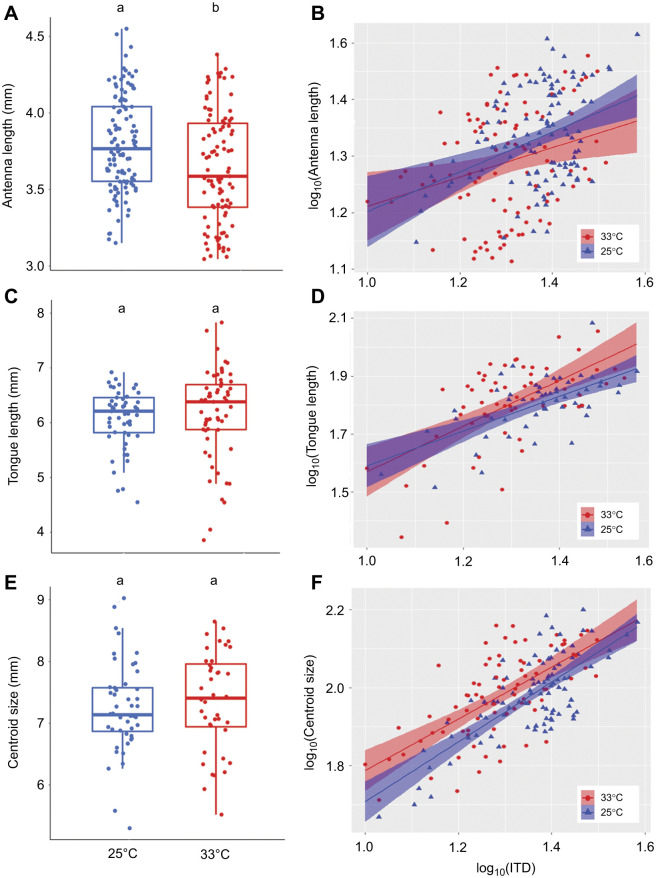
**Impact of developmental temperature on worker bumblebee morphological traits and scaling relationship between body size and these traits.** (A) Antennal length (*n*=210; LMM; *P*=0.01). (B) Scaling relationship between body size (IDT) and antennal length (*n*=208, LMM; *P*=0.117). (C) Tongue length (*n*=112, LMM; *P*=0.61). (D) Scaling relationship between body size and tongue length (*n*=112, LMM; *P*<0.001). (E) Wing centroid size (*n*=175, LMM; *P*=0.87). (F) Scaling relationship between body size and wing centroid size (*n*=175, LMM; *P*<0.001). Different letters at the top of the boxplots indicate significant differences. Each dot represents the measurement for one individual. For A, C and E, the first, second and third horizontal bars represent, respectively, the first quartile, the median and the third quartile. The two vertical bars represent the minimum and maximum, without considering the outliers.

**Fig. 3.**
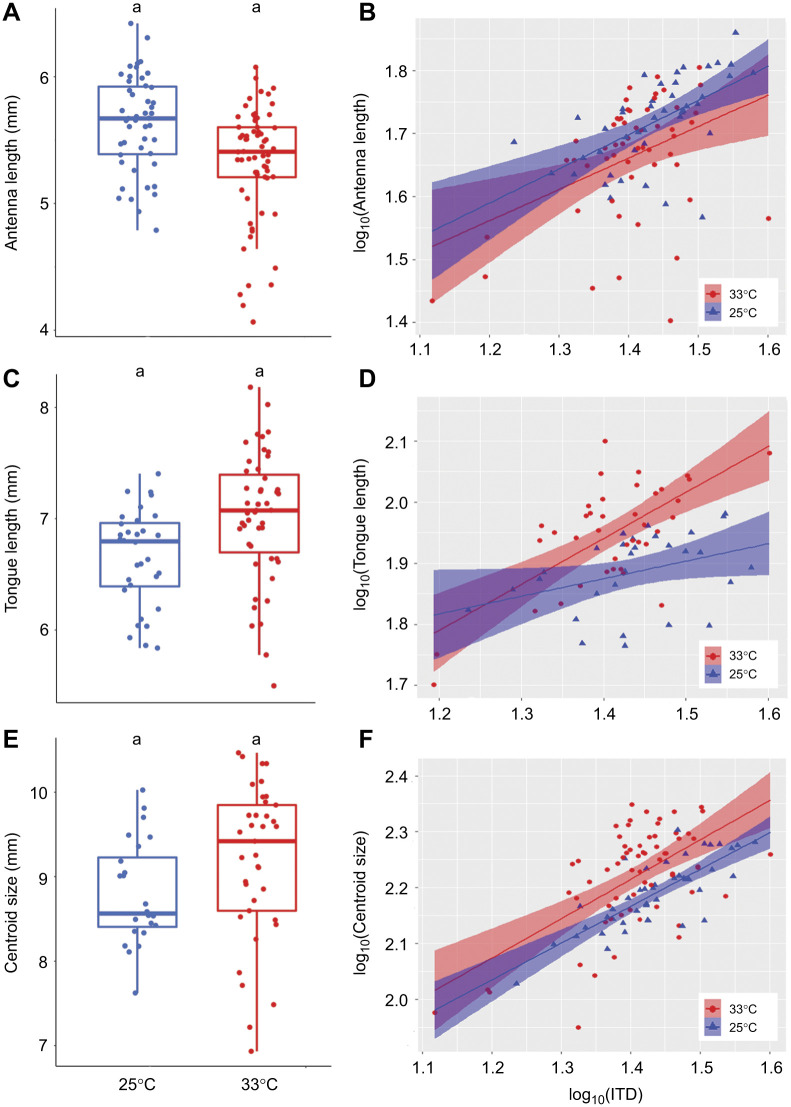
**Scaling relationship between male body size and the different morphological traits.** (A) Antennal length (*n*=111, LMM; *P*<0.001). (B) Scaling relationship between body size and antennal length (*n*=100, LMM; *P*=0.02). (C) Tongue length (*n*=79, LMM; *P*=0.35). (D) Scaling relationship between body size and tongue length (*n*=68, LMM; *P*=0.02). (E) Wing centroid size (*n*=120, LMM; *P*=0.138). (F) Scaling relationship between body size and wing centroid size (*n*=109, LMM; *P*<0.001). Different letters at the top of the boxplots indicate significant differences. Each dot represents the measurement for one individual. For A, C and E, the first, second and third horizontal bars represent, respectively, the first quartile, the median and the third quartile. The two vertical bars represent the minimum and maximum, without considering the outliers.

The model that best explained the variation in antennal length (ΔAICc=8.89 with the next best candidate model, Table S2) included temperature, session and colony for workers (*r*^2^=0.46, Table 1) and only temperature and colony for males (ΔAICc=1.88 with the next best candidate model, Table S3; *r*^2^=0.12, Table 2). Exposure to 33°C during development reduced the antennal length of workers (*P*=0.01; Fig. 2A). However, temperature did not significantly affect the antennal length for males (*P*=0.3; Fig. 3A). The random factors ‘colony’ and ‘session’ explained, respectively, 11.2% and 45.3% of the variance that remained in the residuals for workers. The random factor ‘colony’ explained 43.4% of the variance that remained in the residuals for males. Variance in antennal length for males and workers was not significantly affected by temperature treatment (*P*=0.2 and *P*=0.12, respectively).

The model that best explained the variation in tongue length for workers (ΔAICc=3.66 with the next best candidate model, Table S2) included temperature, session and colony (*r*^2^=0.44; Table 1), and for males (ΔAICc=2.27 with the next best candidate model, Table S3) it included temperature and colony (*r*^2^=0.37; Table 2). Developmental temperature had no effect on the tongue length of either workers (*P*=0.61; Fig. 2C) or males (*P*=0.37; Fig. 3C). The random factors ‘session’ and ‘colony’ explained, respectively, 36.8% and 14.8% of the variance that remained in the residuals for workers. The random factor ‘colony’ explained 32.6% of the variance that remained in the residuals for males. The variance in tongue length increased significantly with temperature for workers (*P*=0.022), but was not significantly affected by temperature in males (*P*=0.106).

Finally, the model that best explained the variation of wing size included temperature and colony for both workers (ΔAICc=2.25 with the next best candidate model, Table S2; *r*^2^=0.18; Table 1) and males (ΔAICc=2.33 with the next best candidate model, Table S3; *r*^2^=0.33; Table 2). Developmental temperature did not significantly affect the wing size of either workers (*P*=0.87; Fig. 2E) or males (*P*=0.169; Fig. 3E). The random factor ‘colony’ explained 18.6% and 29.97% of the variation that remained in the residuals, respectively, for workers and males. While wing size variance significantly increased with temperature in males (*P*=0.042), it was not significantly affected by temperature treatment in workers (*P*=0.525).

### Allometric components of the morphological variation

Antennal length increased significantly with ITD for both males and workers (*P*<0.001; Figs 2B and 3B, Tables 1 and 2; ΔAICc=7.8 and 6.74, respectively with the next best candidate model, Tables S2 and S3), although the relationship was hypoallometric, meaning that the antennae of the larger bumblebees were proportionally smaller than the antennae of smaller bumblebees. For both workers and males, temperature did not significantly impact the allometric slopes (Figs 2B and 3B) as this variable was not included in the best model, suggesting that developmental temperature does not affect the allometric relationship between ITD and antennal length in males and workers.

Tongue length increased significantly with ITD in both males and workers (*P*<0.001; Figs 2D and 3D, Tables 1 and 2; ΔAICc=3.57 and 1.51, respectively with the next best candidate model, Tables S2 and S3), although the relationship was hypoallometric, meaning that the tongues of the larger bumblebees were proportionally shorter than the tongues of smaller bumblebees. For both sexes, temperature treatment had a significant effect on the allometric slopes (Figs 2B and 3B): the allometric slope of tongue length was steeper at 33°C than at 25°C for both workers (*P*=0.025, Fig. 2D) and males (*P*<0.001, Fig. 3D), suggesting that at 33°C, the hypoallometric trend was not as strong as at 25°C.

Wing size increased significantly with ITD in both workers and males (*P*<0.001; Figs 2F and 3F, Tables 1 and 2; ΔAICc=4.17 and 4.84, respectively with the next best candidate model, Tables S2 and S3), although the relationship was hypoallometric, meaning that the wings of the larger bumblebees were proportionally smaller than the wings of smaller bumblebees. For workers, temperature did not significantly affect the allometric slopes (Fig. 2F), as this variable was not included in the best model. For males, temperature treatment was included in the best model but it did not significantly affect the allometric scaling (*P*>0.05). These results suggest that developmental temperature does not affect the allometric relationship between ITD and wing size either in males or in workers.

## DISCUSSION

The main goal of this study was to assess how developmental temperatures affect different morphological traits associated with foraging efficiency in bumblebees. While exposure to a higher than optimal temperature during development led to smaller body size and antennae length in workers, it did not have any significant effect on wing size or tongue length. Exposure to 33°C during development affected the tongue length variance of workers and the wing size variance of males. For each trait, there was a significant hypoallometric relationship with body size. The allometric analysis also suggested that, for both castes, tongue length was affected by developmental temperature. Indeed, the tongue lengths of larger bumblebees were proportionally smaller than those for smaller bumblebees for both castes, and this trend was even stronger at 25°C. This corroborates previous studies showing that allometric coefficients can be impacted by temperature (Stevens, 2004; Shingleton et al., 2009).

A reduction in body size at higher developmental temperatures is commonly observed in insects and is known as the temperature–size rule or TSR (Atkinson, 1994; Angiletta and Dunham, 2003), although there is no single or simple explanation for this rule (see Verberk et al., 2020, for a detailed review of the potential mechanisms). Among the mechanistic hypotheses that have been proposed, van der Have and de Jong (1996) suggested that the TSR is related to different temperature sensitivities of growth rate (i.e. increase of mass through time) and development rate (i.e. life stage differentiation through time). Indeed, DNA replication, associated with cell differentiation, and thus development rate, is more sensitive to temperature than protein synthesis, which is associated with growth (van der Have and de Jong, 1996). In this context, individuals reach their mature stage faster than they gain weight (i.e. faster development rate than growth rate). Additional mechanisms exist to explain TSR; for example, the model of von Bertalanffy (1960) and Perrin (1995) suggests that growth duration is not directly affected by temperature. In their model, higher temperature increases the growth rate, and the growth stops when the rate of anabolism balances the rate of catabolism. Thus, if temperature enhances catabolism more than anabolism, balance is achieved sooner in development, at a smaller body size. It is interesting to note that among terrestrial insects with a dry mass of >100 mg (i.e. like many bumblebees), the TSR is not as common as in smaller insects (Horne et al., 2015; Verberk et al., 2020). A considerable number of papers highlight that some larger terrestrial insects do not follow the TSR, or even show that they follow the opposite trend. Still, the TSR has regularly been observed among bees (e.g. Radmacher and Strohm, 2011; Guiraud et al., 2021). Part of the relationship between developmental temperature and body size could be linked to juvenile hormone (Radmacher and Strohm, 2010; 2011). Higher temperatures increase the rate of juvenile hormone clearance by enzymes, which reduces the development time and leads to smaller body size at pupation (Radmacher and Strohm, 2010; 2011). While the TSR is a plastic response to higher developmental temperatures, a smaller body size in bumblebees could be adaptive as it would reduce vulnerability to overheating (Heinrich, 1976). However, a smaller body size can also correlate with decreased foraging distance (Greenleaf et al., 2007) or a lower rate of foraging (Spaethe and Weidenmüller, 2002). Ultimately, it could thus decrease colony performance and affect individual fitness. Other stressors, such as urbanization, can lead to smaller body size and could amplify these effects by acting in synergy (Eggenberger et al., 2019).

For workers, we observed a significant decrease in antennal length at higher developmental temperatures. As the size of sensory organs is typically related to their sensitivity, this reduction in size would suggest that the antennae in these individuals were less sensitive to stimuli (e.g. Riveros and Gronenberg, 2010), which would have a detrimental effect on both foraging and resource detection. Interestingly, we did not observe any changes in mean tongue length or wing size, suggesting that they are fairly resilient to the tested developmental temperatures and may be highly constrained in order to be functional. While the impact of developmental temperatures on bumblebee tongue length has never been tested before in laboratory conditions, a previous study observed that bumblebee males that developed at an elevated temperature had shorter wings (Gérard et al., 2018b). In this previous study, microcolonies – i.e. a colony which includes fewer than 5 workers producing only males – were used rather than full colonies as in the present study. Microcolonies do not have the thermoregulation capabilities of a full colony, which could alter their resilience to stressful temperatures and may explain why short wings were observed at elevated temperature, whereas we did not see this effect in full colonies. Our results also suggest that the mean size of the wings and tongue length are more robust to high developmental temperatures than the mean body size or antennal length, but exactly why this is the case and what the functional consequences are remain to be explored. This is even more intriguing because, for the trait variance, the tongue length of the workers and the wing size of the males were both affected by the high developmental temperature. An increase of trait variance is common under stressful conditions (Hoffmann and Hercus, 2000; Gérard et al., 2022b). It can be detrimental if the morphological trait moves further away from its optimum (Ghalambor et al., 2007), but can also be advantageous under fluctuating conditions, to allow new phenotypes to be selected (Badyaev, 2005).

Allometric differences in morphological traits are common among insects. In bees, they may be adaptive for utilizing different food resources and have been observed both between workers from different colonies and within populations (Owen and Harder, 1995; Perl et al., 2022). More specifically, changes in the allometric scaling of sensory organs or traits related to movement could affect foraging behaviour (Riveros and Gronenberg, 2010; Peters et al., 2016). While the antennae of larger workers were relatively shorter, we did not observe any significant impact of temperature on the ratio of antennal length to body size, suggesting that, overall, antennal length in workers is tightly coupled to body size no matter what developmental temperature the individual experiences. The antennae of the larger males were comparatively shorter than those of the smaller males, suggesting that smaller males invest more in antennal length, possibly to improve their sensitivity to female pheromones and to minimize the fitness cost of being smaller (Spaethe et al., 2007). Tongue length in both castes increased slower than body size at both developmental temperatures, but this trend was even stronger at 25°C. Thus, in our study, higher temperature (i.e. 33°C) drives the relationship between body size and tongue length closer to isometry. With global warming, this trend could lead either to changes in floral resources that bees forage on or, if changes in floral morphology do not match the changes in tongue length, to a morphological mismatch between tongue length and corolla depth (Gérard et al., 2020), as suggested by a previous study on alpine bumblebees (Miller-Struttmann et al., 2015). The comparison of our results with the study of Miller-Struttmann et al. (2015) is interesting. While they observed that the ratio of tongue length to body size was decreasing even more with climate change, we observed that this relationship was closer to isometry at higher developmental temperatures. Miller-Struttmann et al. (2015) hypothesize that climate change does not directly affect tongue length, but rather that it affects floral resources, and that the changes in floral resources resulted in shorter tongues relative to body size during the last decades. In our study, elevated developmental temperatures per se strongly decreased worker body size, and slightly increased tongue length, resulting in larger tongues relative to body size. Thus, the driver of the plastic response we observed seems to differ from the drivers of the tongue size changes that Miller-Struttmann et al. (2015) measured, which could explain this apparent discrepancy.

We highlighted that the relative size of the wings of both males and workers was smaller with larger body size, and that temperature did not affect the slope of this relationship. Our results corroborate other studies that have highlighted the hypoallometric scaling between wings, even though they noticed that this trend was even stronger under stressful developmental conditions (i.e. reduced access to food) (Tigreros et al., 2013; Helm et al., 2021; Grula et al., 2021). These larger wings relative to smaller body size could allow greater distributional range (Rundle et al., 2007), particularly for migrating species (Sacchi and Hardersen, 2013), as well as better acceleration capacity (Bewaerts et al., 2002). Having larger wings in stressful conditions for smaller insects would thus be adaptive, as it would allow individuals to fly longer distances and potentially give them access to new favourable habitats (Rundle et al., 2007; Flockhart et al., 2017). Yet, the mechanisms underlying bee flight are quite different from those of most insects (Dudley and Ellington, 1990; Altshuler et al., 2005); thus, the relationship between wing morphology and flight performance needs to be studied in more detail in other insect clades like bees, to assess whether the same trends are observed.

Several mechanisms have been highlighted to explain the alteration of allometry by developmental temperature. For example, developmental temperatures impact the number or the size of cells in different body parts (Partridge et al., 1994; Van der Have and de Jong, 1996) or the allocation of resources during development (Bochdanovits and de Jong, 2003), which may explain our results on the allometric component of tongue length. Generally, temperature affects cell size rather than the number of cells (Arendt, 2007). Thus, the decrease in cell size could explain both the smaller morphological traits (for worker body size and antennae) and the changes in allometric scaling (for the tongue of both castes) that we observed at the elevated temperature (see Verberk et al., 2020, for a detailed description of cell size changes related to temperature). Whether these differences would be adaptive or not remains to be tested. At the very least, we can safely assume that smaller morphological traits and body size imply fitness costs, as flight, foraging and sensory abilities of bees are tightly linked to organ size (Spaethe and Weidenmüller, 2002; Kelber et al., 2006; Greenleaf et al., 2007). Yet, it is important to consider that our study was conducted in laboratory conditions and that the next step would be to assess whether our results are congruent in the field; for example, for individuals produced after a particularly warm summer. In our study, the ambient temperature was constant during the whole development, whereas, in the field, it would fluctuate and become colder during the night. In addition, some species nest under the ground, which would provide a better buffer against elevated temperatures than the plastic boxes we used in lab conditions (although ground-nesting species are not necessarily able to maintain their brood at a constant temperature under natural conditions; Gradisek et al., 2023). Temperature fluctuations and/or buffering could reduce the phenotypic effects we observed. As many bumblebee species nest above the ground, investigating how this ecological trait drives changes in morphology is crucial for identifying species that could be more affected by global warming. Another important factor to consider is the functional consequences of the morphological modifications we observed, especially on the changes in flight performance and foraging efficiency, and this would be another important focus for future research in this field.

## Supplementary Material

10.1242/jexbio.245728_sup1 Supplementary information
