# Supplementary material for: Elevated developmental temperatures impact the size and allometry of morphological traits of the bumblebee Bombus terrestris
Source: J Exp Biol. 2023 Apr 19;226(8):jeb245728. doi: 10.1242/jeb.245728 (PMC10263145; doi:10.1242/jeb.245728)
Supplement: Supplementary information [file jexbio-226-245728-s1.pdf]

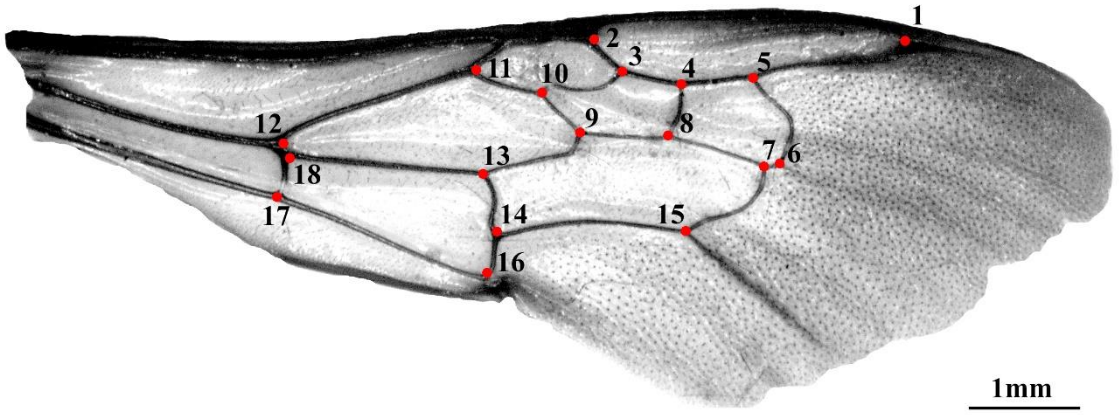

Fig. S1. Right forewing and 18 landmarks of a worker of the buff-tailed bumblebee *Bombus terrestris.*

Table S1. Dataset for each morphological trait

Trait	Sex	Temperature (°C)	N
ITD	Worker	25	180
ITD	Worker	33	163
ITD	Male	25	46
ITD	Male	33	67
Antenna	Worker	25	113
Antenna	Worker	33	97
Antenna	Male	25	46
Antenna	Male	33	61
Tongue	Worker	25	95
Tongue	Worker	33	82
Tongue	Male	25	46
Tongue	Male	33	67
Wing	Worker	25	54
Wing	Worker	33	60
Wing	Male	25	46
Wing	Male	33	67

Journal of Experimental Biology • Supplementary information

Table S2. List of the next best candidate models to predict the impact of temperature on morphological traits and allometric components of workers

Next best candidate model	Predictor	Estimate	Std Error	p-value	ΔAICc
ITD ~ Temperature + (1 | Colony) + (1 | Session)	Intercept	3.647	0.11	0.011	1.34
	Temperature	0.248	0.065	0.006	
Antennae ~ Temperature + (1 | Colony)	Intercept	3.63	0.093	<0.001	8.89
	Temperature	0.248	0.132	0.083	
Tongue ~ Temperature + (1 | Colony)	Intercept	6.077	0.184	<0.001	3.66
	Temperature	0.078	0.258	0.765	
Wing size ~ Temperature + (1 | Colony)	Intercept	7.333	0.185	<0.001	2.25
	Temperature	-0.041	0.253	0.873	
Log_{10}(Antennae) ~ log_{10}(ITD) + log_{10}(ITD):Temperature + (1 | Colony) + (1 | Session)	Intercept	0.657	0.095	0.004	6.74
	Log_{10}(Size)	0.496	0.052	<0.001	
	Log_{10} (Size):temperature	0.022	0.016	0.188	
Log_{10}(Tongue) ~ log_{10}(ITD) + (1 | Colony) + (1 | Session)	Intercept	1.121	0.124	<0.001	1.51
	Log_{10}(Size)	0.516	0.089	<0.001	
Log_{10}(Wing) ~ log_{10}(ITD) + log_{10}(ITD):Temperature + (1 | Colony) + (1 | Session)	Intercept	0.817	0.083	<0.001	4.17
	Log_{10}(Size)	0.908	0.051	<0.001	
	Log_{10} (Size):temperature	-0.033	0.013	0.033	

Journal of Experimental Biology • Supplementary information

Table S3. List of the next best candidate models to predict the impact of temperature on morphological traits and allometric components of males

Model	Predictor	Estimate	Std Error	p-value	ΔAICc
ITD ~ Temperature + (1 | Colony) + (1 | Session)	Intercept	4.103	0.072	<0.001	2.18
	Temperature	0.079	0.121	0.53	
Antennae ~ Temperature + (1 | Colony) + (1 | Session)	Intercept	5.228	0.162	0.01	1.88
	Temperature	0.186	0.202	0.382	
Tongue ~ Temperature + (1 | Colony) + (1 | Session)	Intercept	6.077	0.376	0.029	2.27
	Temperature	0.102	0.194	0.608	
Wing size ~ Temperature + (1 | Colony) + (1 | Session)	Intercept	7.333	0.185	<0.001	2.33
	Temperature	-0.041	0.253	0.873	
Log_{10}(Antennae) ~ log_{10}(ITD) + log_{10}(ITD):Temperature + (1 | Colony) + (1 | Session)	Intercept	1.028	0.134	<0.001	7.8
	Log_{10}(Size)	0.433	0.091	<0.001	
	Log_{10} (Size):temperature	0.01	0.024	0.691	
Log_{10}(Tongue) ~ log_{10}(ITD) + (1 | Colony) + (1 | Session)	Intercept	1.19	0.139	<0.001	3.57
	Log_{10}(Size)	0.515	0.097	<0.001	
Log_{10}(Wing) ~ log_{10}(ITD) + (1 | Colony) + (1 | Session)	Intercept	1.342	0.112	<0.001	4.84
	Log_{10}(Size)	0.61	0.079	<0.001	

Journal of Experimental Biology • Supplementary information
